# Clinical Characteristics of Patients Who Died of Coronavirus Disease 2019 in China

**DOI:** 10.1001/jamanetworkopen.2020.5619

**Published:** 2020-04-10

**Authors:** Jianfeng Xie, Zhaohui Tong, Xiangdong Guan, Bin Du, Haibo Qiu

**Affiliations:** 1 Department of Critical Care Medicine, School of Medicine, Zhongda Hospital, Southeast University, Nanjing, China; 2 Department of Respiratory and Critical Care Medicine, Beijing Institute of Respiratory Medicine, Beijing Chao-yang Hospital, Capital Medical University, Beijing, China; 3 Department of Critical Care Medicine, The First Affiliated Hospital of Sun Yat-sen University, Guangzhou, China; 4 Medical Intensive Care Unit, Peking Union Medical College Hospital and Chinese Academy of Medical Sciences, Beijing, China

## Abstract

This case series describes the characteristics of a cohort of patients who died of coronavirus disease 2019 in China.

## Introduction

The outbreak of coronavirus disease 2019 (COVID-19) has been very severe in China.^1^ As of March 2020, many tens of thousands of patients have had confirmed COVID-19, and cases have been increasing daily.^2^ The mortality is much higher in Wuhan, China, than in other cities.^3^ To understand the characteristics of patients who die of COVID-19, we analyzed 168 patients with COVID-19–induced pneumonia who died.

## Methods

This case series’ study protocol was approved by each local institutional ethics committees. Written informed consent was waived owing to the urgent need to collect data. Data were obtained from 21 hospitals in Wuhan, China. Demographic, comorbidity, and respiratory support data for 168 patients who died of COVID-19 between January 21 to 30, 2020, in these hospitals were collected. All patients were diagnosed as having COVID-19 according to World Health Organization guidance.^3^ All patients underwent nucleic acid testing by reverse transcription–polymerase chain reaction testing, and their results were positive for COVID-19. Categorical variables were described as numbers (proportions) and continuous variables were described as medians and interquartile ranges (IQRs). Data were analyzed from February 8 to February 10, 2020.

## Results

Of 168 patients who died, 126 (75.0%) were men. The median (IQR) age was 70 (64-78) years, and 161 patients (95.8%) were older than 50 years. The age distribution of men and women patients is presented in the Figure, A. Most patients (125 patients [74.4%]) had 1 or more comorbidities. The Figure, B, presents the distribution of chronic comorbidities among these patients. Hypertension was the most common comorbidity (84 patients [50.0%]), followed by diabetes (42 patients [25.0%]), and ischemic heart disease (31 patients [18.5%]).

**Figure.**
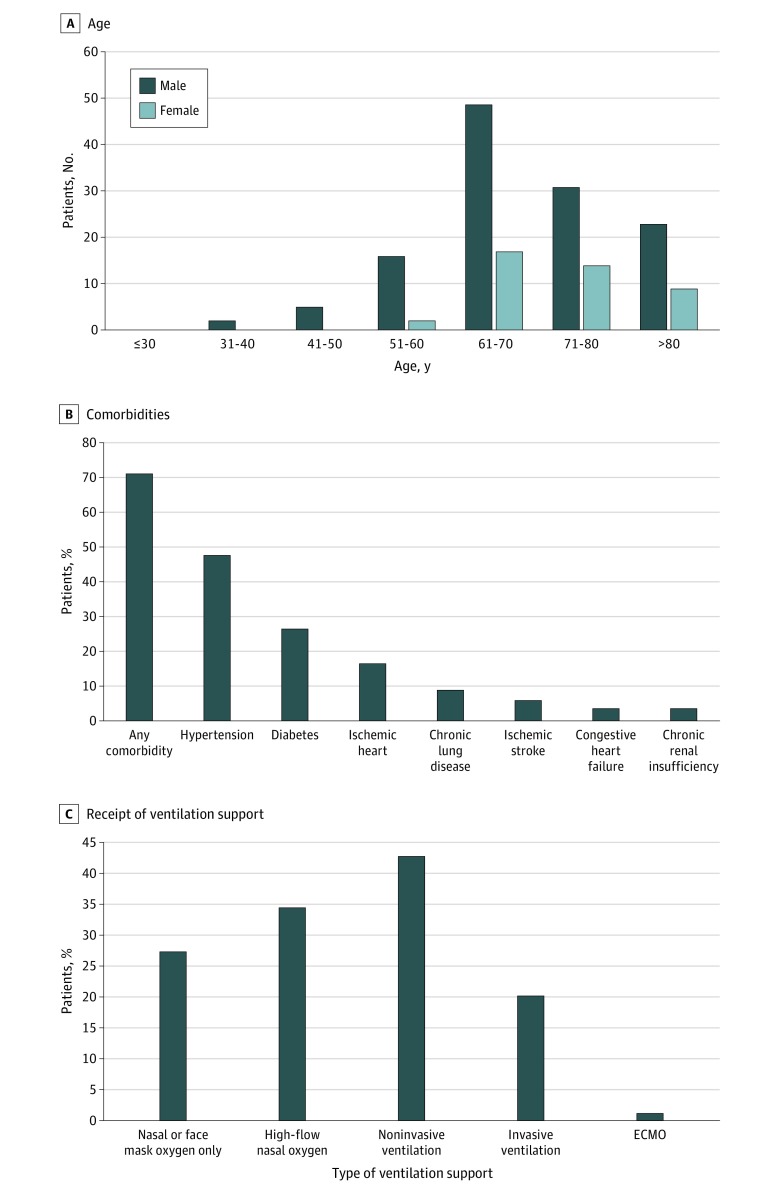
Characteristics of Patients Who Died of Coronavirus Disease 2019 A, Age distribution of patients stratified by age and sex. B, Chronic comorbidities distribution among patients. C, Proportion of patients who received ventilation support. ECMO indicates extracorporeal membrane oxygenation.

All patients received oxygen therapy during their hospital stay. However, 46 patients (27.4%) only received nasal or face mask oxygen before they died (Figure, C). In addition, approximately one-third of patients (58 patients [34.5%]) received high-flow nasal oxygen therapy, and 72 patients (42.9%) received noninvasive ventilation. However, only 34 patients (20.2%) were intubated and received invasive mechanical ventilation, and 2 patients (1.2%) received extracorporeal membrane oxygenation treatment. Age was not a factor associated with intubation.

## Discussion

The results of this case series show that only approximately one-fifth of patients who died of COVID-19 received invasive mechanical ventilation and further aggressive respiratory support prior to death, indicating that many patients had delayed intubation. A 2015 study showed that delayed intubation after the failure of high-flow nasal oxygen or noninvasive ventilation for patients with moderate and severe respiratory failure was associated with increased mortality.^4^ Additionally, approximately 27% of patients only received nasal or face mask oxygen treatment before they died. Several reasons may explain this low proportion. First, some patients with severe hypoxemia did not have other symptoms, such as shortness of breath or dyspnea, also called *silent hypoxemia.* Second, the lack of enough invasive mechanical ventilators is an important reason that would prevent patients from receiving intubation. Third, a medical team that is not dominated by intensivists may not receive critical care training; therefore, they may be uncertain on the timing for when a patient requires intubation.

Another interesting finding of this case series is that hypertension was the most common chronic comorbidity among patients who died. A previous 2020 case series^1^ also reported a higher rate of hypertension among patients with COVID-19 who were admitted to intensive care units than among patients with COVID-19 who were not admitted to intensive care units. However, hypertension usually is not an independent risk factor associated with mortality in patients with sepsis.^5^ According to a study from earlier this year,^6^ severe acute respiratory syndrome coronavirus 2 infects the lungs through the angiotensin-converting enzyme II receptor. Further research is needed to find the mechanism of COVID-19. In addition, clinical studies are also needed to confirm whether angiotensin-converting enzyme inhibitors and angiotensin receptor blockers could be beneficial for patients with COVID-19.

Our study has some limitations. One limitation is that our data were from patients who died during late January 2019, and they may not be representative of later cases of COVID-19.

This case series found that delayed intubation was common in the early stage of the COVID-19 epidemic in Wuhan. Potential reasons for the delay include lack of invasive mechanical ventilators and lack of specific clinical training for respiratory support.
