# Error in Discussion

**DOI:** 10.1001/jamanetworkopen.2020.8147

**Published:** 2020-05-08

In the research letter titled “Clinical Characteristics of Patients Who Died of Coronavirus Disease 2019 in China,”^1^ published April 10, 2020, there was an error in the Discussion. The second sentence of the third paragraph of the Discussion section should have read “One limitation is that our data were from patients who died during late January 2020, and they may not be representative of later cases of COVID-19.” The article has been corrected.
